# Supplementary material for: G-Protein-Coupled Receptors Mediate Modulations of Cell Viability and Drug Sensitivity by Aberrantly Expressed Recoverin 3 within A549 Cells
Source: Int J Mol Sci. 2023 Jan 1;24(1):771. doi: 10.3390/ijms24010771 (PMC9820968; doi:10.3390/ijms24010771)
Supplement: Supplementary file 1 [file ijms-24-00771-s001.zip › ijms-2028362-supplementary.pdf]

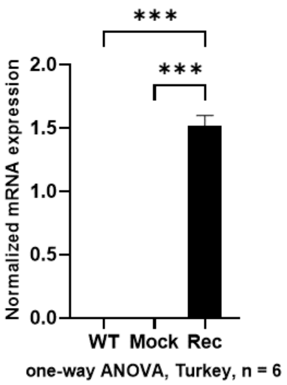


**Supplemental Figure S1 mRNA expression of Rec among WT, A549 MOCK and A549 Rec.**

2D cultured A549 (WT), A549 MOCK and A549 Rec were subjected to qPCR analysis to estimate the expression of mRNA in *Rec*. All experiments were performed in triplicate for each experimental condition. Data are presented as the arithmetic mean ± standard error of the mean (SEM). ****P*<0.005 (ANOVA followed by a Turkey's multiple comparison test).

**<u>Primer;</u>**

Primer1 5'-GCGTGTTTTCATCGTCTGGA-3',

Primer2 5'-CCTTCTCCCTCTACGACGTG-3',

Probe 5'-/56-FAM/ACTTCATTC/ZEN/TTGCTGATGGTCCCGT/3IABkFQ/-3'

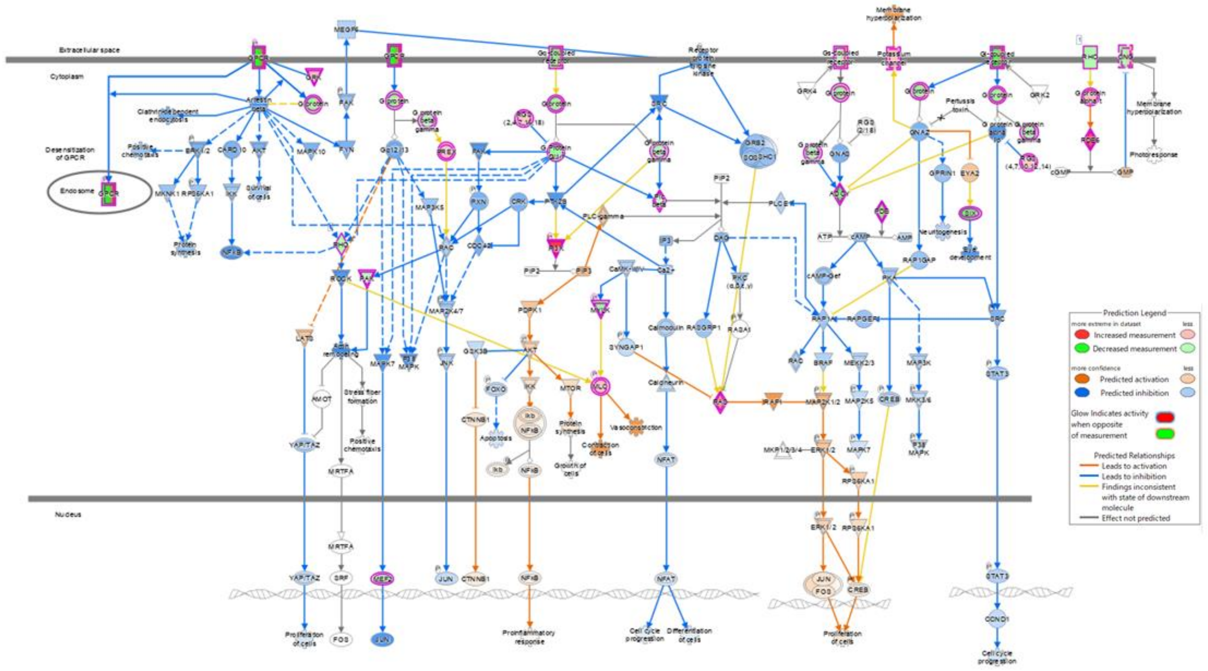


**Supplemental Figure S2 Canonical network of G-protein coupled receptor (GPCR) signaling**

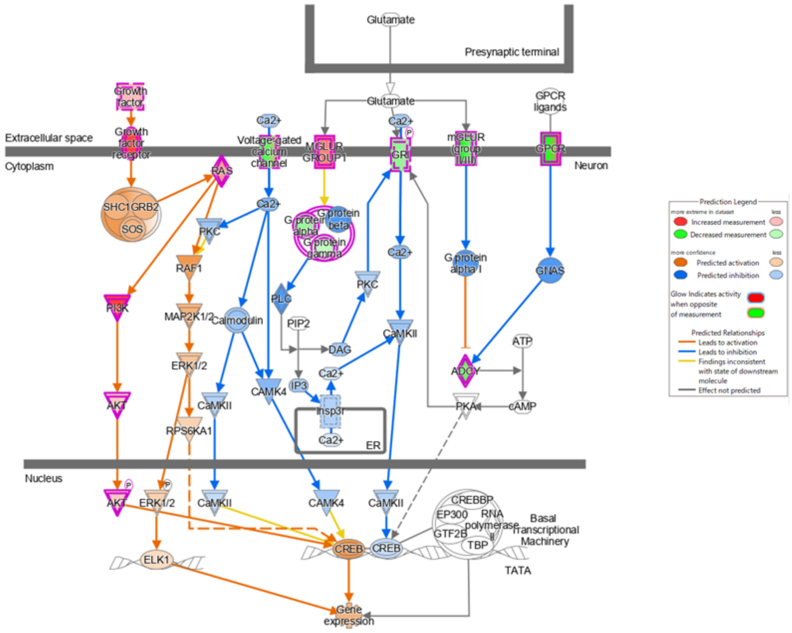


**Supplemental Figure S3 Canonical network of CREB signaling**

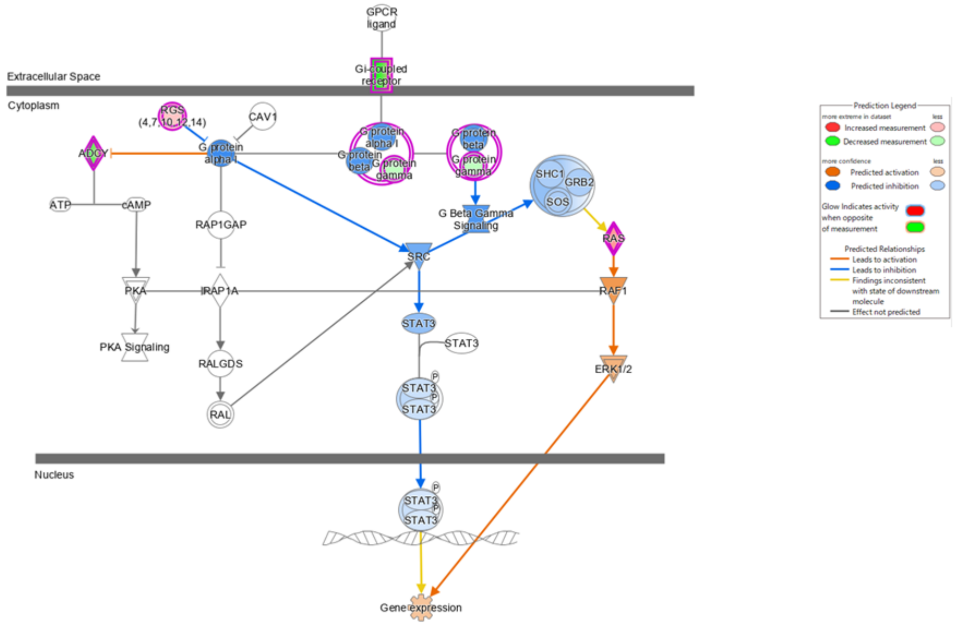


**Supplemental Figure S4 Canonical network of Gia mediated signaling**

**Supplemental Table S1.** Sequence reads and mapping information of A549 Mock and A549 Rec.

| Sample Name | Total reads | Trimmed Reads | Mapped reads | Mapping rate |
|---|---|---|---|---|
| A549_Rec1 | 35258092 | 34284440 | 33781100 | 98.53% |
| A549_Rec2 | 42631970 | 41192892 | 40546696 | 98.43% |
| A549_Rec | 34299918 | 33567356 | 32962995 | 98.20% |
| A549_Mock1 | 36606010 | 35473726 | 34947741 | 98.52% |
| A549_Mock2 | 35675928 | 34243940 | 33723546 | 98.48% |
| A549_Mock | 35126452 | 34364062 | 33782843 | 98.31% |

**Supplemental Table S2.** List of the up-regulated or down-regulated DEGs (FDR adjusted p>0.05 and $q < 0.08$) between A549 MOCK and A549 Rec.

| gene_sym | log2FoldChange | p-value | q-value | Cellular_Component_Term | Molecular_Function_Term | Biological_Process_Term |
|---|---|---|---|---|---|---|
| RCVRN | 14.73 | <0.001 | <0.001 | perikaryon, dendrite, membrane, photoreceptor inner segment, photoreceptor outer segment, | calcium sensitive guanylate cyclase activator activity, calcium ion binding, | regulation of calcium ion transport, positive regulation of guanylate cyclase activity, phototransduction, visual perception, signal transduction, |
| KIFC1 | 5.04 | >0.001 | 0.003 | mitotic spindle, membrane, microtubule, kinesin complex, microtubule organizing center, early endosome, nucleus, | ATP hydrolysis activity, microtubule binding, ATP binding, microtubule motor activity, | mitotic spindle assembly, cell division, mitotic metaphase plate congression, microtubule-based movement, mitotic sister chromatid segregation, |
| ATF6B | 4.71 | 0.002 | 0.005 | RNA polymerase II transcription regulator complex, protein-DNA complex, Golgi apparatus, endoplasmic reticulum membrane, nucleus, chromatin, | sequence-specific double-stranded DNA binding, cAMP response element binding, protein binding, DNA-binding transcription factor activity, RNA polymerase II-specific, RNA polymerase II cis-regulatory region sequence-specific DNA binding, | ATF6-mediated unfolded protein response, endoplasmic reticulum unfolded protein response, signal transduction, regulation of transcription by RNA polymerase II, |

| | | | | | | |
|---|---|---|---|---|---|---|
| | | | | | transcription cis-regulatory region binding, | |
| OR51E2 | 4.62 | 0.01 | 0.027 | intracellular organelle, early endosome membrane, integral component of membrane, plasma membrane, | signaling receptor activity, olfactory receptor activity, G protein-coupled receptor activity, nuclear steroid receptor activity, | regulation of renin secretion into blood stream, melanocyte proliferation, cellular response to fatty acid, detection of chemical stimulus involved in sensory perception of smell, regulation of blood pressure, regulation of cAMP-mediated signaling, steroid hormone mediated signaling pathway, melanocyte differentiation, cell migration, G protein-coupled receptor signaling pathway, |
| AC073911.3 | 4.55 | 0.015 | 0.026 | | | |
| AC107959.2 | 4.47 | 0.018 | 0.028 | | | |
| AP000240.1 | 4.3 | 0.037 | 0.042 | | | |
| AP001625.2 | 4.28 | 0.033 | 0.04 | | | |
| IL6R-AS1 | 4.1 | 0.01 | 0.023 | | | |
| AC020663.1 | 4 | 0.013 | 0.024 | | | |
| LENG8 | 3.79 | <0.001 | <0.001 | protein-containing complex, nucleus, | protein binding, | |
| BMS1P13 | 3.56 | 0.047 | 0.049 | | | |
| ERMARD | 3.55 | 0.001 | 0.005 | integral component of membrane, endoplasmic reticulum membrane, | | |
| AC027307.3 | 3.53 | 0.018 | 0.029 | | | |
| ZNF251 | 3.32 | >0.001 | 0.002 | nucleus, | metal ion binding, protein binding, DNA-binding transcription factor activity, RNA polymerase II-specific, RNA polymerase II cis-regulatory | hematopoietic stem cell homeostasis, regulation of transcription by RNA polymerase II, |

| | | | | | | |
|---|---|---|---|---|---|---|
| | | | | | region sequence-specific DNA binding, | |
| DPH6-DT | 3.1 | 0.038 | 0.043 | | | |
| AC009065.3 | 2.76 | 0.024 | 0.032 | | | |
| C4B-AS1 | 2.6 | 0.002 | 0.006 | | | |
| MOAP1 | 2.44 | 0.009 | 0.024 | cytosol, mitochondrial outer membrane, mitochondrion, cytoplasm, | ubiquitin protein ligase binding, protein binding, | regulation of autophagosome assembly, extrinsic apoptotic signaling pathway in absence of ligand, apoptotic signaling pathway, regulation of release of cytochrome c from mitochondria, intrinsic apoptotic signaling pathway in response to DNA damage, extrinsic apoptotic signaling pathway via death domain receptors, autophagy, protein insertion into mitochondrial membrane involved in apoptotic signaling pathway, |
| MRM1 | 2.41 | <0.001 | <0.001 | mitochondrial matrix, mitochondrion, | rRNA (guanine) methyltransferase activity, protein binding, RNA binding, | rRNA 2'-O-methylation, rRNA modification, |
| SRMS | 2.2 | 0.027 | 0.032 | extrinsic component of cytoplasmic side of plasma membrane, cytosol, cytoplasm, | ATP binding, protein binding, signaling receptor binding, protein tyrosine kinase activity, | innate immune response, peptidyl-tyrosine autophosphorylation, cell differentiation, peptidyl-tyrosine phosphorylation, regulation of signal transduction, transmembrane receptor protein tyrosine kinase signaling pathway, |
| AC239859.6 | 2.18 | 0.02 | 0.03 | | | |

| | | | | | | |
|---|---|---|---|---|---|---|
| LRRC37A5P | 1.95 | 0.049 | 0.049 | | | |
| ITPK1 | 1.68 | <0.001 | <0.001 | apical plasma membrane, cytosol, | inositol-1,3,4-trisphosphate 5-kinase activity, inositol-1,3,4-trisphosphate 6-kinase activity, inositol tetrakisphosphate 1-kinase activity, isomerase activity, inositol tetrakisphosphate 6-kinase activity, magnesium ion binding, | necroptotic process, inositol phosphorylation, inositol trisphosphate metabolic process, neural tube development, blood coagulation, signal transduction, |
| AC010643.1 | 1.31 | 0.034 | 0.04 | | | |
| CEACAM6 | 1.28 | <0.001 | <0.001 | azurophil granule membrane, anchored component of membrane, apical plasma membrane, cell surface, extracellular space, | protein heterodimerization activity, protein binding, | regulation of anoikis, regulation of endothelial cell-matrix adhesion via fibronectin, regulation of heterotypic cell-cell adhesion, regulation of cell migration, regulation of cell population proliferation, signal transduction, heterophilic cell-cell adhesion via plasma membrane cell adhesion molecules, apoptotic process, |
| AL359198.1 | 1.26 | 0.041 | 0.044 | | | |
| GTF2IP1 | 1.22 | 0.021 | 0.03 | | | |
| EIF3C | 1.16 | 0.012 | 0.025 | eukaryotic 48S preinitiation complex, eukaryotic 43S preinitiation complex, eukaryotic translation initiation factor 3 complex, cytosol, | ribosome binding, translation initiation factor binding, protein binding, RNA binding, | regulation of mRNA binding, regulation of translation, translational initiation, formation of cytoplasmic translation initiation complex, |
| SBNO2 | 1.13 | <0.001 | <0.001 | nucleus, | histone binding, chromatin DNA binding, | cellular response to leukemia inhibitory factor, osteoclast fusion, cellular response to lipopolysaccharide, bone trabecula |

| | | | | | | |
|---|---|---|---|---|---|---|
| ABCC6 | 1.02 | 0.014 | 0.029 | basolateral plasma membrane, integral component of membrane, plasma membrane, endoplasmic reticulum membrane, nucleoplasm, extracellular region, | ATPase-coupled inorganic anion transmembrane transporter activity, ATPase-coupled transmembrane transporter activity, ABC-type glutathione S-conjugate transporter activity, ATP binding, | morphogenesis, regulation of inflammatory response, regulation of transcription by RNA polymerase II, regulation of DNA-templated transcription, osteoclast differentiation, bone mineralization, macrophage activation involved in immune response, response to sodium phosphate, biomineralization, leukotriene transport, transmembrane transport, calcium ion homeostasis, phosphate ion homeostasis, ATP metabolic process, response to magnesium ion, cellular phosphate ion homeostasis, inorganic diphosphate transport, ATP transport, gene expression, response to xenobiotic stimulus, visual perception, |

| gene_sym | log2FoldChange | p-value | q-value | Cellular_Component_Term | Molecular_Function_Term | Biological_Process_Term |
|---|---|---|---|---|---|---|
| IGSF23 | -4.74 | 0.007 | 0.051 | integral component of membrane, | | |
| AC090673.1 | -4.64 | 0.023 | 0.063 | | | |
| ARR3 | -4.53 | 0.032 | 0.056 | synapse, cytoplasm, photoreceptor inner segment, photoreceptor outer segment, | phosphoprotein binding, protein binding, opsin binding, G protein-coupled receptor binding, | visual perception, signal transduction, G protein-coupled receptor internalization, regulation of protein phosphorylation, |

| | | | | | | |
|---|---|---|---|---|---|---|
| AC007405.3 | -4.38 | 0.021 | 0.06 | | | |
| GPR158 | -4.37 | 0.026 | 0.058 | integral component of membrane, plasma membrane, | G protein-coupled receptor activity, | protein localization to plasma membrane, G protein-coupled receptor signaling pathway, |
| AL009178.3 | -4.35 | 0.03 | 0.057 | | | |
| AL590006.1 | -4.3 | >0.001 | 0.009 | | | |
| AC096720.2 | -4.24 | 0.034 | 0.051 | | | |
| INTS9-AS1 | -4.22 | 0.035 | 0.051 | | | |
| AC007216.2 | -4.1 | 0.042 | | | | |
| MAGED4B | -4.02 | 0.006 | 0.061 | nucleus, | protein binding, | regulation of transcription by RNA polymerase II, |
| AC096751.2 | -3.78 | 0.025 | 0.058 | | | |
| AL512506.1 | -3.68 | 0.036 | 0.049 | | | |
| AC104113.1 | -3.65 | 0.036 | 0.048 | | | |
| CDC37P1 | -3.08 | 0.039 | 0.049 | | | |
| LINC02256 | -3.05 | 0.02 | 0.064 | | | |
| RN7SKP173 | -2.99 | 0.046 | 0.048 | | | |
| SMN2 | -2.98 | 0.021 | 0.062 | Gemini of coiled bodies, perikaryon, neuron projection, cytoplasmic ribonucleoprotein granule, SMN-Sm protein complex, SMN complex, axon, Z disc, nuclear body, Cajal body, cytosol, cytoplasm, nucleoplasm nucleus, | protein binding, RNA binding, | nervous system development, DNA-templated transcription termination, spliceosomal snRNP assembly, spliceosomal complex assembly, |
| SNORA71A | -2.86 | 0.024 | 0.059 | nucleolus, | | RNA processing, |
| LUNAR1 | -2.83 | 0.01 | 0.057 | | | |
| AP001992.1 | -2.78 | 0.037 | 0.047 | | | |

| | | | | | | |
|---|---|---|---|---|---|---|
| UBR7 | -2.69 | 0.019 | 0.07 | | ubiquitin protein ligase activity, zinc ion binding, | protein ubiquitination, |
| AC108448.3 | -2.66 | 0.042 | 0.049 | | | |
| AC067930.5 | -2.53 | 0.049 | 0.049 | | | |
| LY6E | -2.48 | 0.015 | 0.07 | anchored component of membrane, plasma membrane, extracellular region, | acetylcholine receptor inhibitor activity, protein binding, | regulation of signaling receptor activity, regulation of viral entry into host cell, cell surface receptor signaling pathway, |
| AC011330.1 | -2.39 | >0.001 | 0.009 | | | |
| SMIM11A | -2.33 | 0.007 | 0.054 | integral component of membrane, | protein binding, | |
| AC009185.1 | -2.27 | 0.033 | 0.052 | | | |
| ZNF365 | -2.25 | 0.043 | 0.048 | centrosome, cytoplasm, | metal ion binding, protein binding, | dendrite arborization, regulation of DNA strand resection involved in replication fork processing, dendritic spine morphogenesis, regulation of oligodendrocyte differentiation, cerebellar molecular layer morphogenesis, regulation of neuron projection development, regulation of double-strand break repair via homologous recombination, telomere maintenance, telomere maintenance, |
| SPTIN5 | -2.06 | 0.028 | 0.058 | cell division site, septin complex, microtubule cytoskeleton, synaptic vesicle, septin ring, plasma membrane, | molecular adaptor activity, GTP binding, protein binding, structural molecule activity, GTPase activity, | regulation of synaptic vesicle exocytosis, cytoskeleton-dependent cytokinesis, social behavior, adult behavior, regulation of exocytosis, |

| | | | | | | |
|---|---|---|---|---|---|---|
| | | | | | | synaptic vesicle targeting, protein localization, |
| AL590644.1 | -2.06 | 0.032 | 0.057 | | | |
| CUBN | -2.05 | 0.033 | 0.053 | extracellular exosome, receptor complex, lysosomal lumen, microvillus membrane, brush border membrane, extrinsic component of external side of plasma membrane, endocytic vesicle, apical plasma membrane, clathrin-coated pit, cytosol, Golgi apparatus, endoplasmic reticulum, endosome, lysosomal membrane, | protein homodimerization activity, cargo receptor activity, signaling receptor activity, cobalamin binding, protein binding, calcium ion binding, | establishment of localization in cell, lipoprotein transport, response to bacterium, cobalamin metabolic process, cholesterol metabolic process, receptor-mediated endocytosis, tissue homeostasis, |
| GCOM2 | -2.02 | 0.036 | 0.048 | | | |
| DHRS11 | -1.97 | 0.003 | 0.032 | extracellular region, | cycloeucalenone reductase activity, 17-beta-hydroxysteroid dehydrogenase (NADP+) activity, 17-beta-ketosteroid reductase activity, 17-beta-hydroxysteroid dehydrogenase (NAD+) activity, estradiol 17-beta-dehydrogenase activity, 3-keto sterol reductase activity, nucleotide binding, | estrogen biosynthetic process, steroid biosynthetic process, |
| NAPEPLD | -1.93 | 0.019 | 0.072 | extracellular exosome, membrane-bounded organelle, photoreceptor outer segment membrane, Golgi apparatus, early endosome, cytoplasm, | N-acetylphosphatidylethanolamine-hydrolysing phospholipase activity, N-acylphosphatidylethanolamine-specific phospholipase D activity, protein binding, bile acid binding, zinc ion binding, | regulation of brown fat cell differentiation, N-acylphosphatidylethanolamine metabolic process, regulation of inflammatory response, host-mediated regulation of intestinal |

| | | | | | | |
|---|---|---|---|---|---|---|
| | | | | nucleoplasm, nuclear envelope, Golgi membrane, | | microbiota composition, phospholipid catabolic process, temperature homeostasis, retinoid metabolic process, |
| PHF1 | -1.93 | 0.035 | 0.05 | site of double-strand break, centrosome, cytoplasm, nucleoplasm, nucleus, | histone methyltransferase binding, metal ion binding, methylated histone binding, protein binding, chromatin binding, DNA binding, transcription corepressor binding, | regulation of histone H3-K27 methylation, cellular response to DNA damage stimulus, regulation of DNA-templated transcription, chromatin organization, |
| AC134775.1 | -1.8 | 0.032 | 0.063 | | | |
| HERC2P8 | -1.74 | 0.024 | 0.06 | | | |
| COLQ | -1.74 | 0.03 | 0.057 | anchoring junction, collagen-containing extracellular matrix, synaptic cleft, neuromuscular junction, extracellular matrix, cell junction, plasma membrane, extracellular space, basement membrane, collagen trimer, | heparin binding, protein binding, extracellular matrix structural constituent, | establishment of protein localization to membrane, skeletal muscle acetylcholine-gated channel clustering, extracellular matrix organization, regulation of synaptic assembly at neuromuscular junction, acetylcholine catabolic process in synaptic cleft, |
| PRRT1B | -1.73 | 0.043 | 0.049 | integral component of membrane, | | |
| WISP1 | -1.64 | 0.009 | 0.056 | extracellular matrix, cytosol, extracellular space, | heparin binding, protein binding, integrin binding, | regulation of wound healing, regulation of cell death, bone development, regulation of inflammatory response, regulation of smooth muscle cell proliferation, regulation of fat cell differentiation, glucose homeostasis, regulation of Wnt signaling pathway, regulation |

| | | | | | | |
|---|---|---|---|---|---|---|
| | | | | | | of smooth muscle cell migration, cell-cell signaling, signal transduction, cell adhesion, regulation of cytokine production, |
| SNORA71C | -1.63 | 0.036 | 0.05 | nucleolus, | | RNA processing, |
| DCHS1 | -1.6 | 0.038 | 0.048 | apical part of cell, catenin complex, integral component of membrane, adherens junction, | cadherin binding, calcium ion binding, | cell-cell adhesion via plasma-membrane adhesion molecules, cochlea development, protein localization to plasma membrane, condensed mesenchymal cell proliferation, digestive tract development, ossification involved in bone maturation, post-anal tail morphogenesis, hippo signaling, adherens junction organization, neurogenesis, neural tube development, pattern specification process, multicellular organism development, cell-cell junction assembly, cell migration involved in endocardial cushion formation, mitral valve formation, branching involved in ureteric bud morphogenesis, cell morphogenesis, |
| AGGF1P2 | -1.57 | 0.046 | 0.049 | | | |
| CCHCR1 | -1.55 | 0.017 | 0.072 | cytosol, centriole, nucleus, | protein binding, | cell differentiation, protein export from nucleus, |
| DTNA | -1.52 | 0.02 | 0.069 | anchoring junction, synapse, intermediate filament | zinc ion binding, protein binding, | synaptic signaling, neuromuscular synaptic transmission, chemical |

| | | | | | | |
|---|---|---|---|---|---|---|
| | | | | cytoskeleton, protein-containing complex, cell junction, plasma membrane, cytoplasm, nucleoplasm, | | synaptic transmission, striated muscle contraction, |
| RRN3 | -1.41 | 0.032 | 0.054 | nucleus, | RNA polymerase I general transcription initiation factor activity, RNA polymerase I core promoter sequence-specific DNA binding, RNA polymerase I core binding, | regulation of DNA-templated transcription initiation, regulation of intrinsic apoptotic signaling pathway, homeostasis of number of cells, fibroblast proliferation, regulation of DNA-templated transcription, ribosome biogenesis, cytoplasm organization, nucleolus organization, transcription initiation at RNA polymerase I promoter, in utero embryonic development, RNA polymerase I preinitiation complex assembly, |
| EPHA10 | -1.34 | 0.002 | 0.026 | receptor complex, neuron projection, plasma membrane, extracellular region, | ATP binding, protein binding, transmembrane-ephrin receptor activity, transmembrane receptor protein tyrosine kinase activity, | ephrin receptor signaling pathway, axon guidance, multicellular organism development, transmembrane receptor protein tyrosine kinase signaling pathway, |
| RASA4CP | -1.34 | 0.042 | 0.049 | | | |
| AL672207.1 | -1.33 | 0.045 | 0.05 | | | |
| STEAP1B | -1.26 | 0.028 | 0.059 | integral component of membrane, plasma membrane, endosome, | protein binding, | |
| SDCCAG8 | -1.24 | >0.001 | 0.007 | photoreceptor cell cilium, ciliary basal body, centriolar | protein binding, | regulation of cilium assembly, tube formation, microtubule organizing |

| | | | | | | |
|---|---|---|---|---|---|---|
| | | | | satellite, cell-cell junction, cytosol, centriole, centrosome, | | center organization, cell projection organization, establishment of cell polarity, centrosome cycle, neuron migration, |
| C1DP1 | -1.17 | 0.029 | 0.058 | | | |
| FPR2 | -1.12 | 0.046 | 0.049 | ficolin-1-rich granule membrane, tertiary granule membrane, specific granule membrane, integral component of membrane, plasma membrane, cytoplasm, | signaling receptor activity, protein binding, scavenger receptor binding, N-formyl peptide receptor activity, G protein-coupled receptor activity, complement receptor activity, amyloid-beta binding, | cellular response to amyloid-beta, regulation of ERK1 and ERK2 cascade, chemotaxis, regulation of phagocytosis, regulation of inflammatory response, regulation of innate immune response, defense response to bacterium, regulation of superoxide anion generation, calcium-mediated signaling, regulation of cytosolic calcium ion concentration, G protein-coupled receptor signaling pathway, cell surface receptor signaling pathway, cell adhesion, complement receptor mediated signaling pathway, regulation of protein phosphorylation, |
| RPL17 | -1.1 | 0.013 | 0.068 | cytosolic ribosome, cytosol, cytoplasm, nucleus, | protein binding, structural constituent of ribosome, RNA binding, | translation, cytoplasmic translation, |
| AC138466.4 | -1.07 | 0.024 | 0.055 | | | |
| PHYHIP | -1.03 | 0.048 | 0.049 | cytoplasm, | protein tyrosine kinase binding, | protein localization, |
